# The Nest Growth of the Neotropical Mound-Building Termite, *Cornitermes cumulans*: A Micromorphological Analysis

**DOI:** 10.1673/031.011.12201

**Published:** 2011-09-19

**Authors:** Marcela I. Cosarinsky

**Affiliations:** Laboratorio de Icnología, Museo Argentino de Ciencias Naturales, Ángel Gallardo 470, (1405) Buenos Aires, Argentina

**Keywords:** Chaco, soil micromorphology, soil-nesting insects

## Abstract

The nests of *Cornitermes cumulans* K. (Isoptera: Termitidae), a very common termite in South American grasslands, display notable morphological transformations during the development of the colony. Young colonies inhabit small subterranean nests that develop into large, conspicuous, epigean mounds, inhabited by very populous colonies. Those macromorphological transformations are accompanied by micromorphological changes occurring gradually in the nest walls. The micromorphological changes during nest development described in the present study expand on previous macromorphological descriptions by explaining the re-organization of the soil components during nest growth. In subterranean nests, walls are composed of piles of lensshaped aggregates of soil material, each one surrounded by a thin organic coating. As the nest grows, mound walls are constructed by disassembling this first lenticular structure and rearranging the materials in a new fabric, where sand grains are loosely distributed among soil microaggregates of organic matter and clay. This is also a temporary construction, because the walls of large nests are composed of a porous mass of sands densely cemented with organic matter and clay in the mound, and a compact mass of the same components in the floor.

## Introduction

Termites of the species *Cornitermes cumulans* (Kollar) (Isoptera: Termitidae) are very common in grasslands of the Chaco region, South America. Its nests are very conspicuous, sub-conical shaped hard soil mounds. The different stages of the nest development were described and three stages were recognized: 1) a subterranean nest, recently founded, 2) a nest partly subterranean, showing a small mound above the soil surface, inhabited by a growing colony, and 3) a large epigean nest, inhabited by a mature colony (Grassé 1958). Fontes (1998) reported polycalic nests, composed of a large mound connected to minor subterranean units called calies.

Two types of growth were distinguished in termite nests: 1) growth by addition and 2) growth by reorganization (Noirot 1970, 1977). In the first case, the new elements are added to the old ones without any modification of the previous construction. The constructions are never remodeled except for the connections to the new parts. The nest of *Cubitermes fungifaber* is a good example of this type of growth (Noirot and NoirotTimothée 1962). In the nests that grow by reorganization, the pre-existing structures are modified while the nest is enlarged. This is the case in *C. cumulans* and other termite species such as *Macrotermes bellicosus* (Noirot 1970) that have nests with a central portion for rearing the larvae, named the hive or habitacle, surrounded by a very resistant wall. The growth of the hive necessarily involves the destruction of the inner part of the wall, and the enlargement of the wall is made not only by the addition of new material on its surface, but also by the remodeling of the remnant wall.

In a previous work the author described important micromorphological differences in different regions of epigean nests of *C. cumulans*, including the peripheral and inner walls, the hive, and the subterranean region (Cosarinsky 2003). These observations showed that a great plasticity occurred in the building process of the different parts of the nest, which is probably related to their specific function. In addition, particular micromorphological features were observed in nests located on different types of soil, with different availability of building soil materials.

This work extends the study of the nest micromorphology of *C. cumulans* to its ontogenetic development. As in previous works, the soil thin section methodology is successfully applied herein to reveal the building materials and how they are distributed in the nest walls. The aim of this work is to investigate if macromorphological nest transformations, from an initial subterranean position to an epigeous structure, include micromorphological changes. Likewise, this micromorphological analysis contributes to deepen the understanding of the reorganization growth process described by Noirot (1970) and is the starting point for further interesting experimental investigations about the relationship between the nest physiology and the micromophology of the nest walls.

## Materials and Methods

### Area description and sampling

The nest micromorphology was studied in the mound-building termite *C. cumulans* using the methodology of soil thin sections commonly employed in Pedology. Nests and surrounding soil were sampled at the Reserva Ecológica E1 Bagual (26° 10′S, 58° 56′W) located in the humid eastern Chaco region, Province of Formosa, Argentina. Scattered nests were located in a distinct area of the Reserva characterized by highlands with a particular sandy soil and grassy vegetation dominantly composed of *Imperata brasiliensis* (chajapé) and *Elionurus muticus* (espartillo). Before this area became protected in 1985, it was under cultivation of cotton and sorghum (Maturo et al. 2005). Three nests showing large, medium, and small mounds were sampled to compare their micromorphology. The sample set was constrained to three colonies by several factors: 1) very few nests of *C. cumulans* occurred at this site located on the same type of soil, 2) the area was the only site of the Reserva where this species was found, 3) their sampling would cause great nest and colony destruction, and 4) the area was under ecological protection.

**Figure 1.**
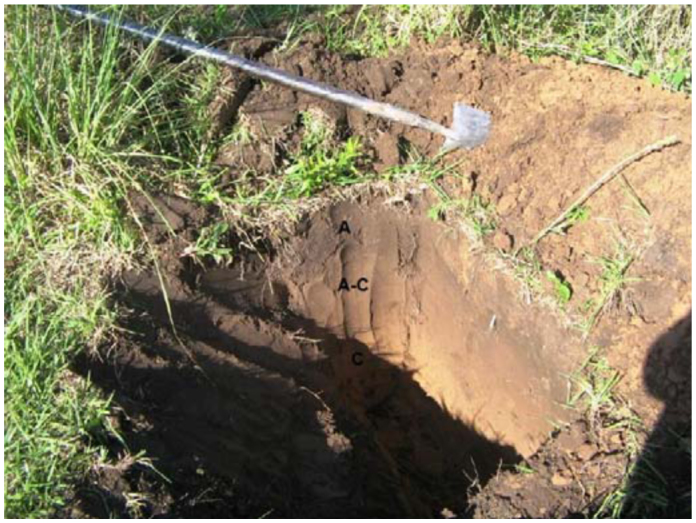
Soil profile where the termite nests were located (entic Hapludoll). A, A–C and C—soil horizons. High quality figures are available online.

The sampled nests were separated each other by 20–30 m, but were located in the same soil type. This is a very important factor to consider in micromorphological comparisons because the nest micromorphology depends on the available soil material employed in the nest building (Cosarinsky 2003). Based on morphological data, the surrounding soil profile was determined as an entic Hapludoll (Keys to Soil Taxonomy, USDA-NRCS, 11^th^ Edition, 2010, http://soils.usda.gov/technical/classification/tax_keys/); a sandy, reddish mollisol, displaying a simple profile composed of horizons A (0– 35 cm), A–C (30–60 cm), and C (60–100 cm or more) (Figure 1). The selected nests were not polycalic; each was characterized by a single, isolated structure.

**Figure 2.**
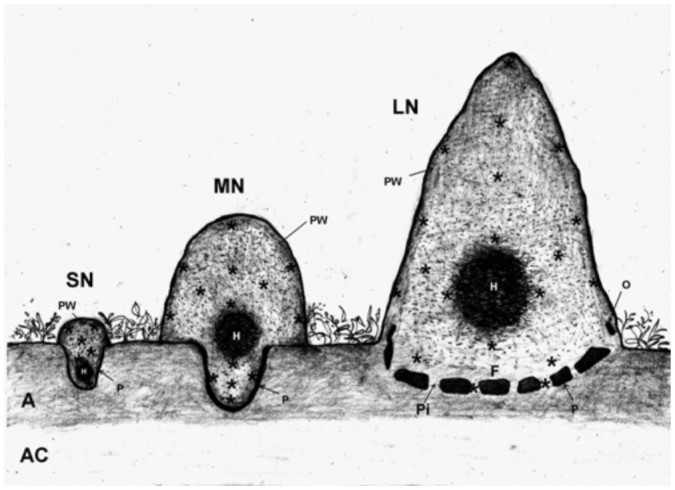
Schematic sections of small, medium and large nests of *Cornitermes cumulans* and profile of the surrounding soil (not in scale). LN, MN and SM—large, medium and small nests respectively; PW— peripheral wall; H—hive; F—floor; Pi—pillar; O—opening; P— “paraecie”; A and A–C—soil horizons; asterisk(*)—nest sample. High quality figures are available online.

In order to identify their stage of development, the three nests were vertically sectioned and their internal morphology observed and compared with the different stages of *C. cumulans* nests described by Grassé (1958), Fontes (1998), and Sanchez et al. (1989). According to those descriptions, young colonies inhabit subterranean nests showing a distinct central region, the hive, displaying an organic carton structure. At this early stage, the nests are entirely surrounded by an empty space, the “paraecie”, which separates its peripheral wall from the surrounding soil. Gradually the nests grow upwards and develop an epigean portion: the mound. The hive moves up from its earlier hypogean location to a central location in the mound. In a more mature stage, the nest shows a conspicuous hypogean portion surrounded by the paraecie and a mound lying directly on the soil surface. The hypogean region gradually decreases, and finally the colonies inhabit a very large epigean nest supported by a minor subterranean portion or nest floor. It is a wide platform entirely separated from the soil by the paraecie, which at this stage connects to the exterior by many openings regularly located around the base of the mound. Following these descriptions, three different stages of development were recognized in the nests sampled (Figure 2).

Field samples of the nests, mounds, and subterranean regions were taken without disturbing their soil structure. Peripheral walls and crossing galleries were sampled from the top to the base, removing 5–8 cm wide horizontal cylindrical sections of material using a narrow shovel with a cutting edge. The galleries situated in the interior of the mounds were sampled, including those located close to the hive. In the large nest, samples of the floor and pillars were also taken. The carton walls of the hives were not sampled because they were exclusively composed of fecal matter (Cosarinsky 2003). Figure 2 shows the location of the nest samples. The surrounding soil of each nest was excavated to a depth of one meter and samples were taken from each horizon to compare the soil micromorphology with the micromorphology of the termite constructions.

### Methodology of soil thin sections

Thin sections were slices of nest walls and soil 30 µm in thickness prepared from undisturbed samples after impregnation with a blue stained, polyester resin (Murphy 1986). They were qualitatively analyzed employing a petrographic microscope. Most micromorphological features were observed under transmitted plain light, whereas the isoand anisotropism of the materials and the birefringence fabrics of the fine material were observed under polarized light. The nomenclature used in micromorphological descriptions and comparative tables was taken from Stoops (2003) and adapted to termite soil nests. The frequency and porosity of coarse components were estimated by a visual system (Fitzpatrick 1984; Bullock et al. 1985).

## Results

### Nests characteristics

**Figure 3.**
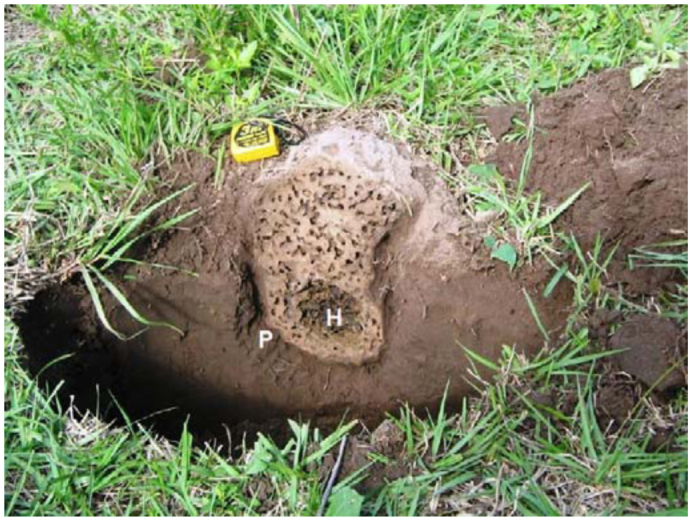
Small nest of *Cornitermes cumulans* (stage 1). Vertical section (H—hive; P—“paraecie”). Size of yellow object: 6 cm. High quality figures are available online.

**Small nest, stage 1.** The small nest was mostly subterranean but showed a very small, flat mound standing on the soil surface (Figures 2, 3). It showed an inverted pear shape and measured 33 cm high including the mound and the subterranean portion, reaching a depth of 20 cm in the A horizon. The mound measured 10 cm high and 20 cm in diameter, and showed externally a light colored compact wall 3 cm in thickness. This wall continued underground, dark brown colored and 0.5–1 cm in thickness. It displayed numerous perforations randomly distributed, measuring 3 mm in diameter. In its interior the nest was crossed by an intricate net of convolute galleries and chambers 0.3–1 cm in diameter, not exceeding 0.5 mm at the subterranean portion. The hive was a distinct sub-spherical structure composed of thin, dark brown carton walls, delimiting interconnected, broad chambers. It was centrally located near the bottom of the nest. The entire nest was easily removed from the soil because it was surrounded by an empty space about 1 cm wide.

**Figure 4.**
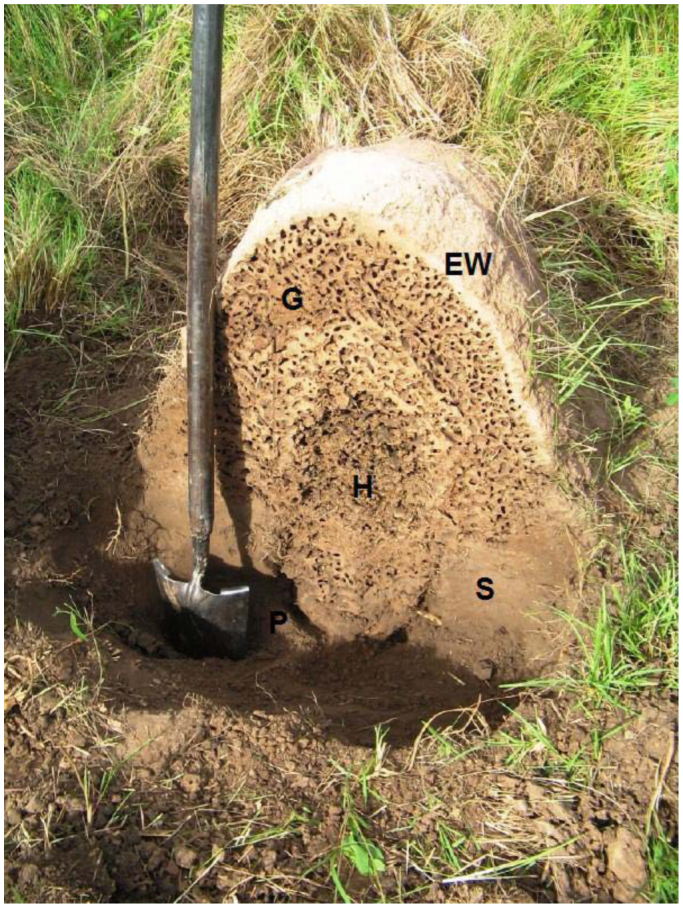
Medium nest of *Cornitermes cumulans* (stage 2). Vertical section of the nest (EW—external wall, G—galleries, H—hive, P— “paraecie”, S—soil). High quality figures are available online.

**Medium nest, stage 2.** The medium nest was composed of a distinct mound and a conspicuous subterranean portion (Figures 2, 4). The mound was 63 cm high and 60 cm in basal diameter. It was domed with a very hard external wall 3 cm thick. The interior of the mound was crossed by an intricate net of galleries and chambers. A close net of galleries 0.5–1 cm in diameter predominated in the periphery, while interior galleries enlarged forming many broad, round, irregular-shaped chambers. The hive was centrally located at the base of the mound and extended downwards into the subterranean portion. The subterranean portion of the nest was located below the central part of the mound base. It was semi-ovoid shaped, narrowing downwards and reaching a depth of 30 cm in the A horizon. The subterranean nest was separated from the soil by an empty space about 2 cm wide. This space entirely surrounded the peripheral subterranean wall and did not connect with the exterior.

**Figure 5.**
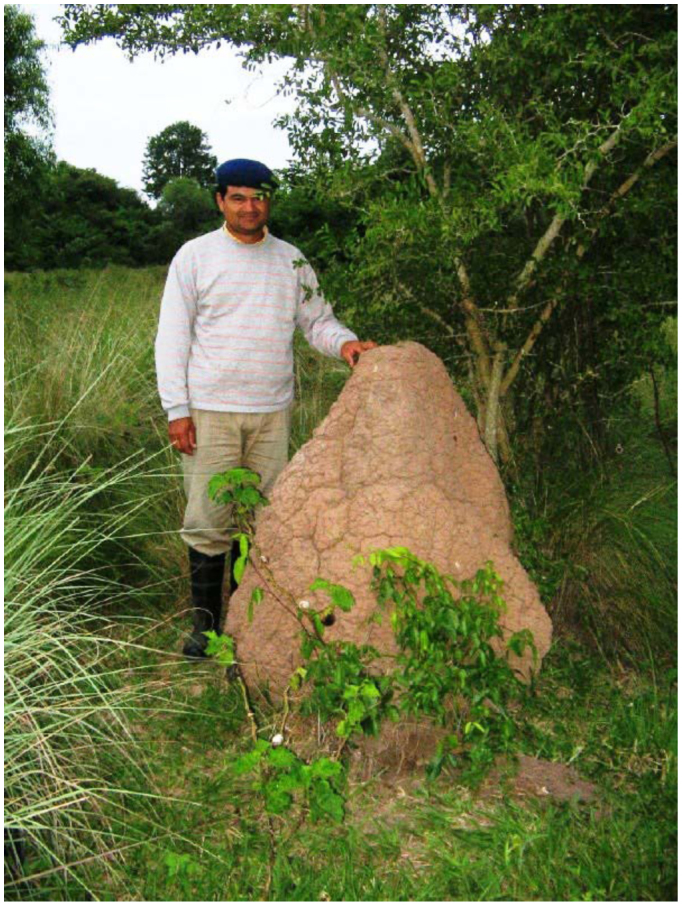
Large nest of *Cornitermes cumulans* (stage 3). Exterior view of the mound located in the field. High quality figures are available online.

**Figure 6.**
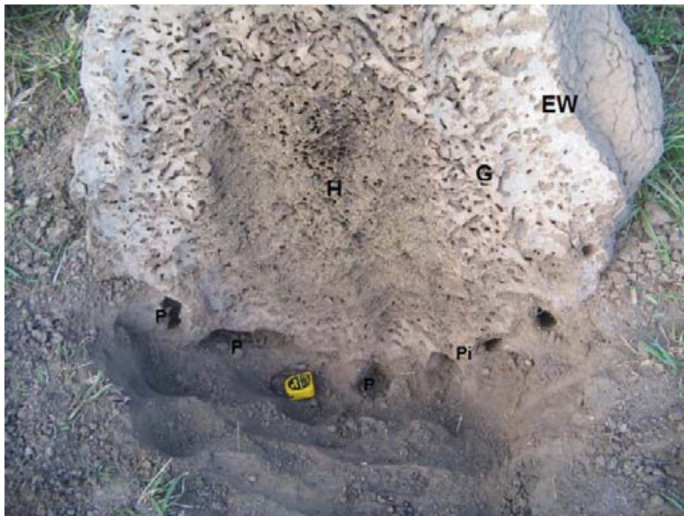
Large nest of *Cornitermes cumulans* (stage 3). Vertical section of the base of the mound and subterranean portion of the nest (H—hive; G—galleries; P—“paraecie”; Pi—pillar). Size of yellow object: 6 cm. High quality figures are available online.

**Large nest, stage 3.** The large nest showed a high, sub-conical mound and a minor subterranean portion (Figures 2, 5, 6). The mound measured 128 cm high and 110 cm in basal diameter. It was conical with a very hard external wall 3–5 cm thick. The interior of the mound was composed of a similar net of galleries and chambers to that observed in the medium mound, along with a large ovoid hive. The subterranean portion was located in the A horizon and showed a compact, smooth floor separated from the soil by an empty space ∼ 5 cm wide. Several short and thick pillars crossed this empty space connecting the nests with the soil. Many round openings ∼ 5 cm in diameter were regularly distributed around the base of the mounds at the soil surface level connecting the empty space with the exterior.

The surface of the three nest galleries was coated by a dark brown lining, covered by abundant, reddish spots. The dark carton walls in the hive were densely covered by similar spots. Likewise, the external wall of the small subterranean nest was coated by a dark brown lining covered by minute reddish spots.

### Micromorphological descriptions

The nests of *C. cumulans* and their surrounding soil were composed of the same coarse, very fine to medium sand sized silts and quartz grains measuring 50–350 µm. The fine material in the nests was clay, distributed as anisotropic streaks surrounding grains (granostriated birefringence fabric) or scattered, anisotropic speckles occurring in a mass of fine, amorphous, organic matter (speckled birefringence fabric). In the soil, microaggregates of fine, organic matter were distributed among grains and did not show interference colors (undifferentiated birefringence fabric).

The following descriptions focus on the most distinct features observed in the thin sections taken from the samples of small, medium, and large sized nests of *C. cumulans* and their surrounding soil.

**Figure 7.**
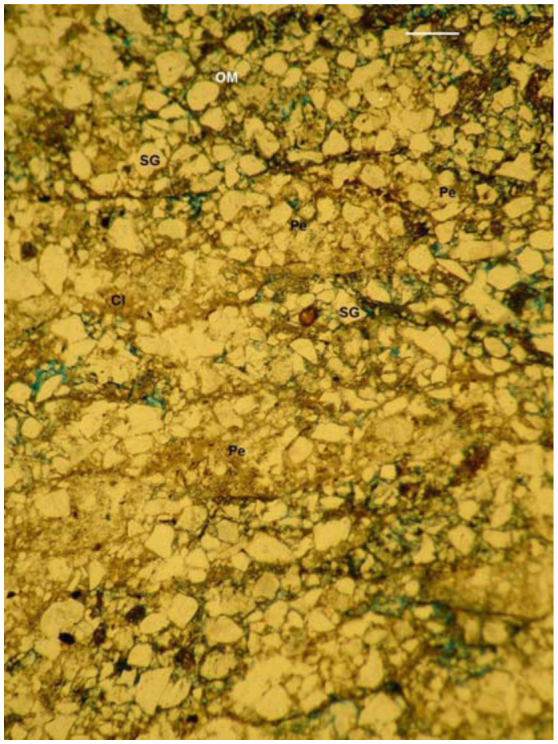
Lenticular microstructure observed in the subterranean walls of the small nest of *Cornitermes cumulans* (stage 1). Scale bar: 250µ. Pe—pellet; SG—sand grain; OM—organic microaggregates; Cl—clay; all voids in blue). High quality figures are available online.

**Small nest, stage 1.** The subterranean walls were composed of piled, elongated lensshaped aggregates (pellets) composed of sand grains, along with clay and organic microaggregates (lenticular microstructure) (Figure 7). Each pellet measured about 375– 500 µm wide and 1–1.5 mm long and was surrounded by a thin organic coating, 25–50 µm thick. The pellets displayed variable aggregation, showing grains densely cemented with abundant clay or weakly adhered by organic microaggregates. Organic coatings lined the galleries with variable thickness. The most distinct ones were 250 µm thick, composed of superposed, planar layers of amorphous, fine organic matter.

**Figure 8.**
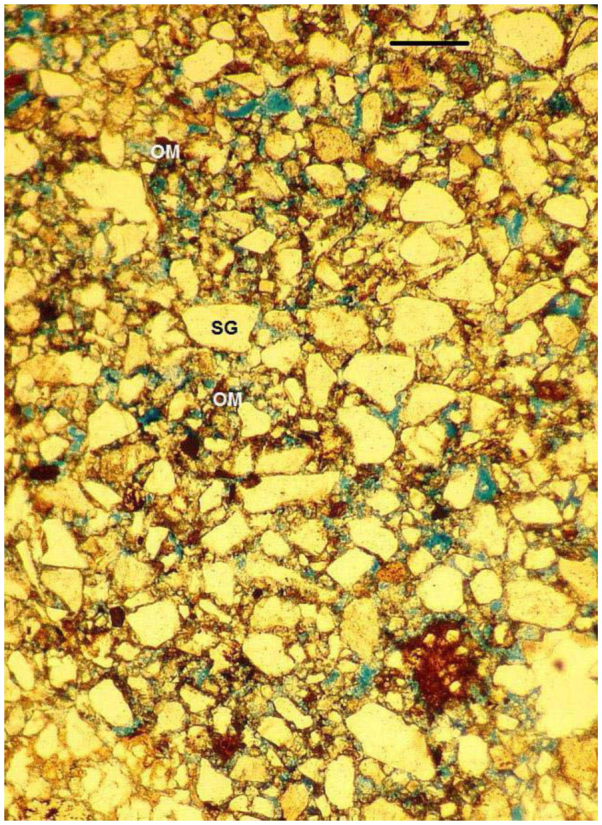
Intergrain microaggregate microstructure observed in the mound walls of the small nest of *Cornitermes cumulans* (stage 1). Scale bar: 250µ. SG—sand grain; OM—organic microaggregates; all voids in blue). High quality figures are available online.

The walls of the little mound above the soil surface displayed a porous structure composed of sand grains loosely aggregated with microaggregates of fine organic matter and clay (intergrain microaggregate microstructure, Figure 8). In some areas, a diffuse lenticular aggregation was observed. Inner galleries showed either a very thin organic coating of 50–75 µm thick or were not coated.

**Medium nest, Stage 2.** The external wall of the mound was composed of areas with sand grains loosely distributed between organic microaggregates (intergrain microaggregate microstructure), alternating with areas displaying a diffuse lenticular aggregation. Many irregular, non-interconnected pores of 0.5–1.5 mm were present. The internal walls of the mound showed the same microstructure as the external wall, but those located in the innermost region close to the hive showed a distinct lenticular microstructure. Galleries and chambers were lined by organic coatings 75–250 µm thick. The subterranean walls surrounding the hive showed areas with lenticular microstructure alternating with areas of intergrain microaggregate microstructure.

**Table 1.**
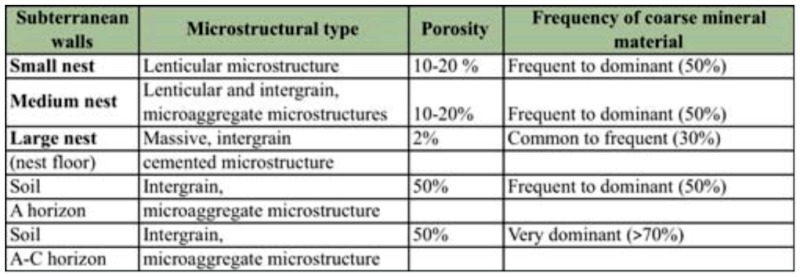
Micromorphological comparisons among subterranean walls of different sized nests and surrounding soil horizons.

**Figure 9.**
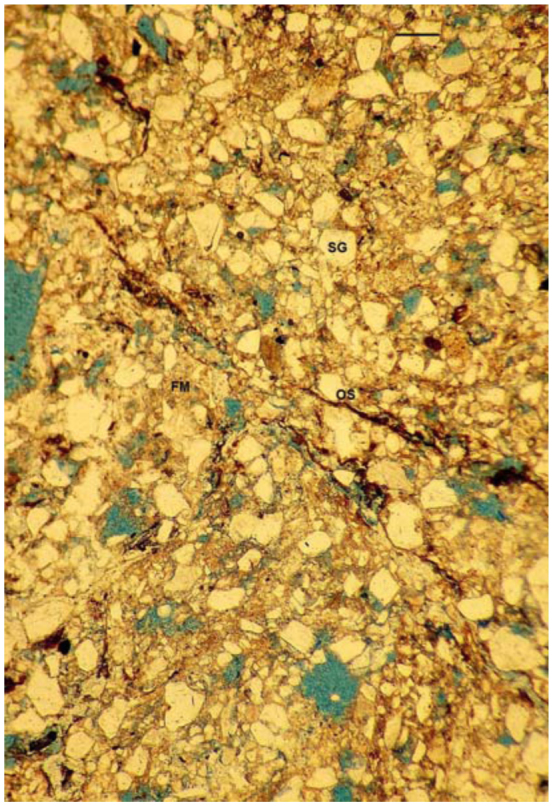
Vughy microstructure observed in the mound walls of the large nest of *Cornitermes cumulans* (stage 3). Scale bar: 250µ. SG— sand grain; FM—fine material (organic matter and clay); OS— organic strand; all voids in blue). High quality figures are available online.

**Figure 10.**
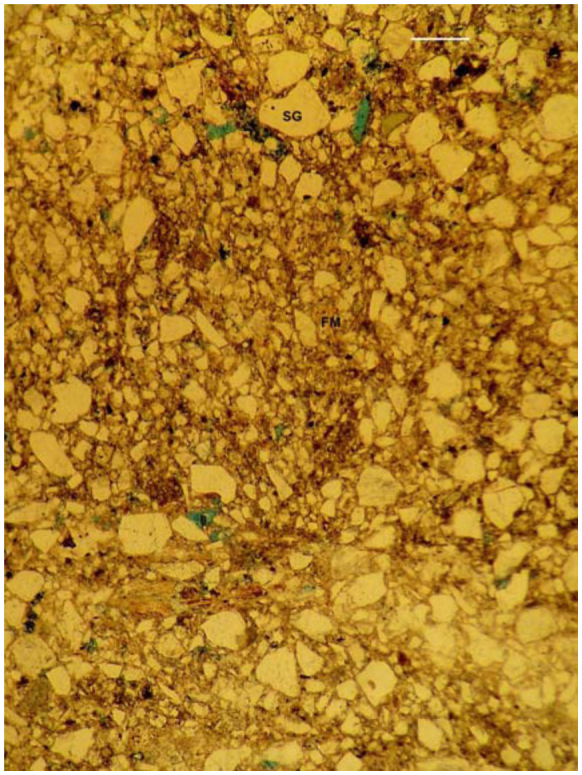
Massive, intergrain cemented microstructure observed in the subterranean floor of the large nest of *Cornitermes cumulans* (stage 3). Scale bar: 250µ. SG—sand grain; FM—fine material (organic matter and clay); all voids in blue). High quality figures are available online.

**Large nest, stage 3.** The external wall of the mound was composed of sands cemented with fine organic matter and clay, interrupted by many non-interconnected and irregular pores (vughs) 50–250 µm in axis (vughy microstructure, Figure 9). Many distinct and randomly oriented organic strands crossed the external wall. Minor areas showed an intergrain microaggregate microstructure. The internal walls showed a similar microstructure, but those located in the innermost region were crossed by wide bands of fine organic matter connecting with the carton walls of the hive. Internal galleries and chambers were lined by organic coatings 75– 350 µm thick. Some galleries were partially infilled with piled, lens-shaped aggregates or soil material displaying a vughy microstructure. The subterranean floor and pillars showed a very compact microstructure composed of sand grains densely cemented by fine organic matter and clay. Very few pores smaller than 75 µm were present (massive, intergrain cemented microstructure) (Figures 10). Pale undulating organic strands crossed the mass, resembling a lenticular structure.

**Surrounding soil.** In the A horizon, sands were distributed among abundant organic microaggregates mixed with clay (intergrain microaggregate microstructure), whereas the A–C horizon was characterized by grains, and few organic microaggregates were placed among them. The C horizon was exclusively composed of loose sands (single grain microstructure).

**Table 2.**
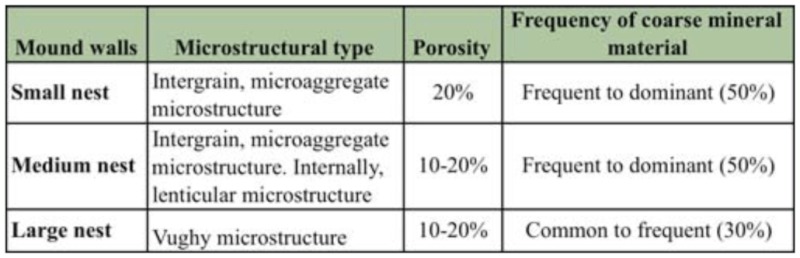
Micromorphological comparisons among the mound walls of different sized nests.

### Micromorphological comparisons

The microstructural types, porosity, and frequency of mineral coarse material of both subterranean and mound walls of nests of different sizes were comparatively summarized in Tables 1 and 2 respectively. The same micro-features observed in the A and A–C horizons were compared with subterranean nest walls in Table 1.

In subterranean walls, microstructure changed from a lenticular structure in the small nest, to a combination of lenticular and intergrain microaggregate structures in the medium nest, and to a massive intergrain cemented structure in the largest nest. The surrounding soil displayed intergrain microaggregate structure. Wall porosity notably decreased from 10–20% in the small and medium nests to only 2% in the largest, whereas soil porosity remained 50%. The frequency of coarse material also decreased from 50% in small and medium nests to 30% in the largest, whereas soil porosity was greater than or equal to 50%.

Microstructure in mound walls changed from intergrain microaggregate structure in the small and medium nest, showing a lenticular structure internally in the latter, to a vughy structure in the largest. A slight decrease of porosity from 20% to 10–20% was shown as nest size increased, and the frequency of coarse material decreases from 50% in the small and medium nests to 30% in the largest.

## Discussion

During the nest growth of *C. cumulans,* gradual micromorphological changes occured in accompaniment with notable architectural variations, revealing a particular behavior of reorganization in the different building materials during nest enlargement and remodeling.

Subterranean walls in the small nest were composed of piles of fitted, lens shaped aggregates (pellets). Their composition is soil sand grains cemented with clay or weakly bound by organic microaggregates, each surrounded by a thin organic coating. This type of microstructure is hereafter called “lenticular microstructure.” The same structure was described for nests of other termites as “massive, pelletai structure” or simply “pelletai structure.” An identical structure was described in nests of *Cortaritermes fulviceps* (Cosarinsky and Roces 2007; Cosarinsky 2004a), *Nasutitermes aquilinus* (Cosarinsky 2005), *Amitermes laurensis, Microcerotermes nervosus, Tumulitermes pastinator* (Lee and Wood 1971), *Drepanotermes rubriceps* (Sleeman and Brewer 1972), and *Macrotermes subhyalinus* (Mermut et al. 1984). In the mound, the microstructure deeply remarkably changes to a very porous structure, where sands are weakly adhered by organic microaggregates. This microstructural type is named “intergrain microaggegate microstructure.” The medium nest shows alternated lenticular and intergrain microaggregated microstructures. Intergrain microaggregate structure prevails in external walls, whereas the lenticular structure prevails interiorly in the walls located close to the hive.

The microstructure differs notably in the large nest. The mound walls show a porous structure composed of sands cemented with fine organic matter mixed with clay. Pores are irregular shaped vughs; hence this structural type is called vughy microstructure. In contrast, pores are very small and few in the subterranean walls of the floor, displaying a massive microstructure also composed of cemented sands.

The frequency of coarse wall components also differs in nests of different sizes. In both small and medium nests it did not differ from the surrounding A horizon and was lower than the A–C horizon, where these termites are supposed to collect the building material. In the large nest, frequency of coarse components was notably lower than the surrounding soil horizons. Similarly, the nest of another mound-building termite, *C. fulviceps*, located at the same Reserva but in alfisol soils, displayed a low frequency of coarse wall components compared with the A horizon (Cosarinsky and Roces 2007).

After the nuptial flight, pairs of de-alates choose the soil to begin the excavation of the nest. They do not excavate randomly in the soil, but instead prefer a substrate enriched with humus (Miranda Lima et al. 2006; Menzel and Diehl 2010). They collect particles from different soil depths and deposit them in mounds, so that content of fine materials like organic carbon and clay are higher in mounds than in adjacent soils (Lal 1988; Black and Okwakol 1997; Ohkuma 2003). High clay content in termite mounds is probably due to their preference for finer soil particles as a cementing material (Lee and Wood 1971; Peres Filho et al. 1990; Donovan et al, 2001). However, it is unknown if termites select particles or if the soil undergoes a physical fractioning through their gut (Lee and Wood 1971; Donovan et al. 2001; Jouquet et al. 2002). In contrast to these observations, no differences in clay content were found between adjacent soil and termite mounds located in natural grasslands in southern Brazil (Kaschuk et al. 2006). The authors explained that in nests located in such clayey soils, a higher sand content might provide better conditions for draining as well as humidity and aeration control.

The addition of clay and organic carbon to the nest was revealed in this study by micromorphological features. Clay addition was shown by the clay birefringence fabrics observed in the three nests, while no birefringence fabric was observed in the soil, where clay birefringence was masked by the humus. Many mound-building termites use saliva to moisten the soil particles carried in the buccal cavity (Noirot and Darlington 2002). When termites remove and mix the soil particles with saliva, clay particles probably decant from suspension in saliva as thin, anisotropric streaks or speckles arranged around the sand grains, displaying granostriated or a speckled birefringence fabrics (Aylmore and Quirk 1960; van Olphen 1977).

The addition of organic carbon was revealed by the presence of organic wall coatings lining the external wall of the small subterranean nest and the inner galleries of all the nests studied. Its layered structure suggests a fecal origin (Lee and Wood 1971; Sleeman and Brewer 1972). Similar coatings were described in nests of many other termite species, such as *Cortaritermes fulviceps* (Cosarinsky and Roces 2007), *Termes saltans* (Cosarinsky 2004b), *Microcerotermes nervosus, Nasutitermes exitiosus* (Lee and Wood 1971), and several Australian species (Sleeman and Brewer 1972). In the large nest, many organic strands were observed crossing the walls of the mound. Their width and composition suggests they are traces of old, organic coatings that lined disused galleries infilled with soil material. Similar strands were observed in a nest of *C. cumulans* located in an alluvial sandy mollisol in the Province of Corrientes, Argentina (Cosarinsky 2003).

The morphogenesis of termite mounds was explained by Turner (2000) as the result of the collective movements of soil within the nest. Those movements may be deposition of soil in empty places (building), or alternatively a result of removal and translocation of soil particles from one part of the mound to another. The rainy season brings rapid translocation of wet soil from deep horizons through *Macrotermes* nests to the mound surface. Soil transport through the mound is tied to water transport and plays a fundamental role in the regulation of the colony water balance. In wetter environments, the adequate nest humidity is achieved by a combination of high evaporative conductance of the mound and the active transport into the nest in the form of liquid water carried up in moist soils from below the ground (Turner 2006; Turner et al. 2006). Similar to *Macrotermes* nests, recent constructions are easily recognized in the mounds of *C. cumulans* as patches of wet and crumbly soil, deposited over the mound surface (Cosarinsky 2003, Turner et al. 2006).

The most common biasing mechanisms in mound construction involve gradients of concentrations in respiratory gases (oxygen, carbon dioxide, water vapor) and temperature (Turner 2001; Korb and Linsenmair 1998). There are close interactions between mound architecture and thermoregulation as well as gas exchange and colony size (Korb 2003; Turner 2000, 2001). However, more experimental studies must be performed to investigate how different wall micromorphologies occurring during nest growth influence thermoregulation and gas exchange.

To conclude, the occurrence of a gradual, chronological, micromorphological sequence is suggested for nest construction in *C. cumulans*. The young colony constructs the walls of the subterranean nest by arranging the soil materials as a lenticular fabric. As the colony grows, mound walls are constructed by disassembling of the first lenticular microstructure, and re-arranging the materials in a new, intergrain microaggregate microstructure. However, in the older interior region of the growing mound close to the hive, a distinct lenticular structure still prevails, and traces of lenticular aggregation persist in the external walls. The intergrain microaggregate structure is a temporary construction; as the colony matures and the mound enlarges, it is transformed into new structural types. The final constructions observed in the large nest show a porous microstructure in the mound and a massive microstructure in the floor. Both regions are composed of sands cemented with abundant clay and fine organic matter, and display a lower frequency of coarse component than previous nest stages. Lenticular structure is almost absent in the mature nest, with the exception of some areas of the subterranean floor where pale, undulating organic strands resemble traces of previous lenticular aggregation.

The gradual micromorphological transformations occurring during nest growth are probably related to variable ecological and physiological needs during the development of the colony. The addition of fine organic matter and clays leads to cohesion and stabilization of the construction at every stage. The organic coatings lining every “lenticular brick” and plastering the galleries and the external wall of the subterranean young nest probably help to maintain adequate nest humidity. Porous walls in epigean mounds are expected to provide good ventilation and thermal isolation to the populous colony, while a massive subterranean floor provides enough consolidation to support such heavy mounds. The loose fabric of grains and microaggregates displayed in temporary constructions is expected to aid in rapid and easy handling and re-location of the building materials. But these relationships between micromorphology and function are merely hypotheses that must be demonstrated with physiological experiments employing undisturbed constructions.
